# The dual role of vitamin C in cancer: from antioxidant prevention to prooxidant therapeutic applications

**DOI:** 10.3389/fmed.2025.1633447

**Published:** 2025-08-29

**Authors:** Xiongfeng Cao, Yide Yi, Minjun Ji, Yanfang Liu, Dongqing Wang, Haitao Zhu

**Affiliations:** ^1^Department of Medical Imaging, Affiliated Hospital of Jiangsu University, Zhenjiang, China; ^2^Institute of Medical Imaging and Artificial Intelligence, Jiangsu University, Zhenjiang, China; ^3^Department of Pharmacy, Affiliated Hospital of Jiangsu University, Zhenjiang, China; ^4^Laboratory of Medical Imaging, The First People’s Hospital of Zhenjiang, Zhenjiang, China

**Keywords:** vitamin C, ascorbic acid, cancer prevention, cancer treatment, tumor microenvironment

## Abstract

Vitamin C (VC), a pleiotropic molecule with context-dependent redox properties, exhibits dual roles in cancer biology through dose-dependent mechanisms. While nutritional VC intake demonstrates chemopreventive effects by scavenging carcinogen-induced reactive oxygen species (ROS) and maintaining genomic stability, high-dose intravenous VC acts as a prooxidant to selectively kill tumor cells via ROS-mediated deoxyribonucleic acid (DNA) damage, adenosine triphosphate (ATP) depletion, and HIF-1α degradation. Preclinical studies reveal VC’s ability to reprogram the tumor microenvironment (TME) through collagen hydroxylation-mediated extracellular matrix remodeling, Treg suppression, and enhancement of CD8+ T cell infiltration. Importantly, VC synergizes with conventional therapies by radio-sensitizing hypoxic tumors through oxygen-sparing effects and reversing platinum resistance via glutathione depletion. Early-phase clinical trials corroborate VC’s safety profile and potential to ameliorate chemotherapy-induced fatigue and nephrotoxicity. However, translational challenges persist, including the lack of pharmacokinetic standardization between oral and intravenous routes, tumor-type-specific response heterogeneity, and incomplete understanding of VC’s immunomodulatory dynamics. Emerging strategies integrating VC with checkpoint inhibitors and TME-targeted nano-delivery systems show promise in preclinical models. This review synthesizes mechanistic insights from redox biology and immunometabolism to clinical trial data, proposing a framework for optimizing VC-based combination therapies while addressing critical gaps in biomarker development and dose scheduling. Deciphering the molecular determinants of VC’s context-dependent anticancer effects may accelerate its rational clinical deployment.

## Graphical Abstract

VC for cancer prevention and cancer therapy. VC, vitamin C; TME, tumor microenvironment. 
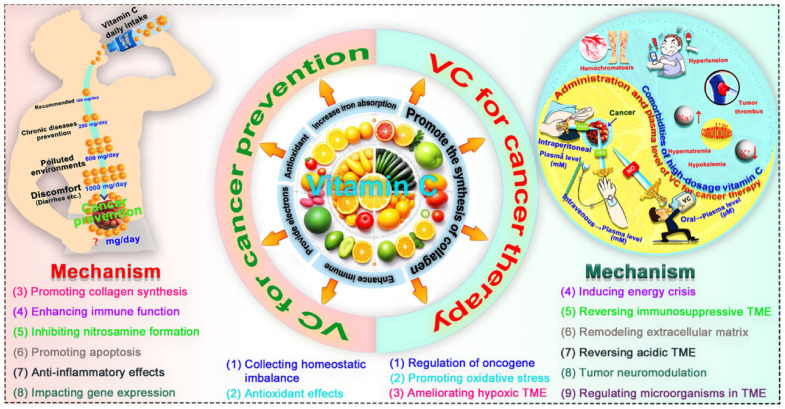

## 1 Introduction

Cancer persists as the second leading cause of global mortality, with epidemiological projections estimating 19.97 million new cases and 9.74 million deaths in 2024 (1). While conventional therapies, including surgical resection, radiotherapy, and chemotherapy, remain cornerstones of cancer management, their efficacy in advanced malignancies remains suboptimal (objective response rates < 40% in metastatic disease) and frequently incurs severe toxicities (grade ≥ 3 adverse events in 45–60% of patients) (2–4). This therapeutic dilemma underscores an urgent need for safer strategies that synergize prevention and treatment paradigms.

Nutraceuticals have emerged as compelling candidates in translational oncology, leveraging their dual capacity to potentiate anticancer effects while mitigating treatment-related toxicity (5, 6). Ascorbic acid (vitamin C, VC) exemplifies this duality through its concentration-dependent redox switching: At physiological levels (50–200 μM), VC acts as a reactive oxygen species (ROS)-scavenging antioxidant that suppresses carcinogenesis by reducing oxidative deoxyribonucleic acid (DNA) damage (7). Conversely, pharmacologic doses (achieving ≥ 0.2 mM plasma concentrations via intravenous administration) trigger prooxidant cascades that selectively target malignant cells through hydrogen peroxide (H_2_O_2_)-mediated responses, including adenosine triphosphate (ATP) depletion, iron-regulated epigenetic reprogramming, anti-apoptotic protein B cell lymphoma 2 (Bcl-2) inhibition, Bax (Bcl-2 associated X protein, a well-known pro-apoptotic mediator) activation, mitochondrial hyperpolarization induction, the cleavage of poly (ADP-ribose) polymerase (PARP, a known apoptotic indicator) and caspases, mainly caspases 3 and 7 as well as targeting hypoxia-inducible factor-1α (*HIF-*α) (8, 9). This concentration-dependent duality positions VC as both a chemopreventive agent and an oxidative stress amplifier for tumor eradication. Emerging preclinical evidence further reveals VC’s pleiotropic modulation of the tumor microenvironment (TME). Through collagen hydroxylase-dependent extracellular matrix remodeling, VC normalizes tumor vasculature to enhance drug delivery while suppressing regulatory T cells (Tregs) and reinvigorating CD8+ T cell cytotoxicity (10). In pancreatic cancer models, VC synergizes with gemcitabine by downregulating matrix metalloprotein 9 (MMP-9) to inhibit metastasis (62% reduction in liver metastases) and depleting glutathione to reverse platinum resistance (11, 12). Contemporary meta-analyses further corroborate its prophylactic value, showing inverse correlations between dietary VC intake and glioma incidence (13). The therapeutic potential of VC was first substantiated in the 1970s when Cameron and Pauling demonstrated prolonged survival in terminal cancer patients receiving high-dose intravenous VC (HDIVC) (10 g/day) (14). These findings have reinvigorated translational interest in optimizing VC’s dual roles, exploiting its redox plasticity for prevention while harnessing prooxidant activity for targeted therapy.

The therapeutic application of VC in oncology has been plagued by persistent controversies, particularly regarding dose-response relationships and administration route efficacy. Seminal randomized controlled trials by Creagan and Moertel administering oral VC (10 g/day) failed to replicate Cameron’s reported survival benefits in terminal cancer patients, casting doubt on VC’s clinical utility (15, 16). This discrepancy was later attributed to pharmacokinetic disparities: oral administration achieves plasma concentrations ≤ 200 μM versus 20 mM through intravenous infusion, a critical threshold for triggering tumor-selective oxidative stress via H_2_O_2_ generation (17–19). Contemporary clinical investigations have refocused on intravenous protocols, with 12 active phase II/III trials evaluating high-dose VC (1–1.5 g/kg) as monotherapy or adjuvant to checkpoint inhibitors/chemotherapy across solid tumors. However, conflicting data persist in three domains: (1) Dose Optimization Paradox: While pharmacokinetic modeling correlates ≥ 15 mM plasma VC with kirsten rat sarcoma viral oncogene (*KRAS*)-mutant tumor regression, lower doses (5–10 mM) paradoxically enhance glutathione synthesis in 32% of glioblastoma cases (20, 21). (2) Metastatic regulation: Preclinical models demonstrate VC’s capacity to suppress epithelial-mesenchymal transition via 10–11 translocation 1 (*TET1*) -mediated MMP-9 downregulation, yet population studies associate supplemental VC with 1.3-fold increased recurrence risk in ER^+^ breast cancer (22, 23). (3) Safety-efficacy balance: Grade ≥ 3 hemolysis occurs in 18% of hematologic malignancy patients receiving 1.5 g/kg VC, contrasting with its proven capacity to reduce chemotherapy-induced fatigue and neuropathic pain (20, 23). To address these challenges, emerging strategies are integrating VC with checkpoint inhibitors and nanotechnology-enabled TME-targeted delivery systems, showing promise in preclinical models.

In this review, we synthesize mechanistic insights from redox biology and immunometabolism with clinical trial data to propose a framework for optimizing VC-based combination therapies (Graphical abstract). We also address critical gaps in biomarker development and dose scheduling, aiming to decipher the molecular determinants of VC’s context-dependent anticancer effects. By doing so, we hope to accelerate the rational clinical deployment of VC in the prevention and treatment of cancer.

## 2 Biological characteristics of VC

### 2.1 Biological properties and functions

**FIGURE 1:**
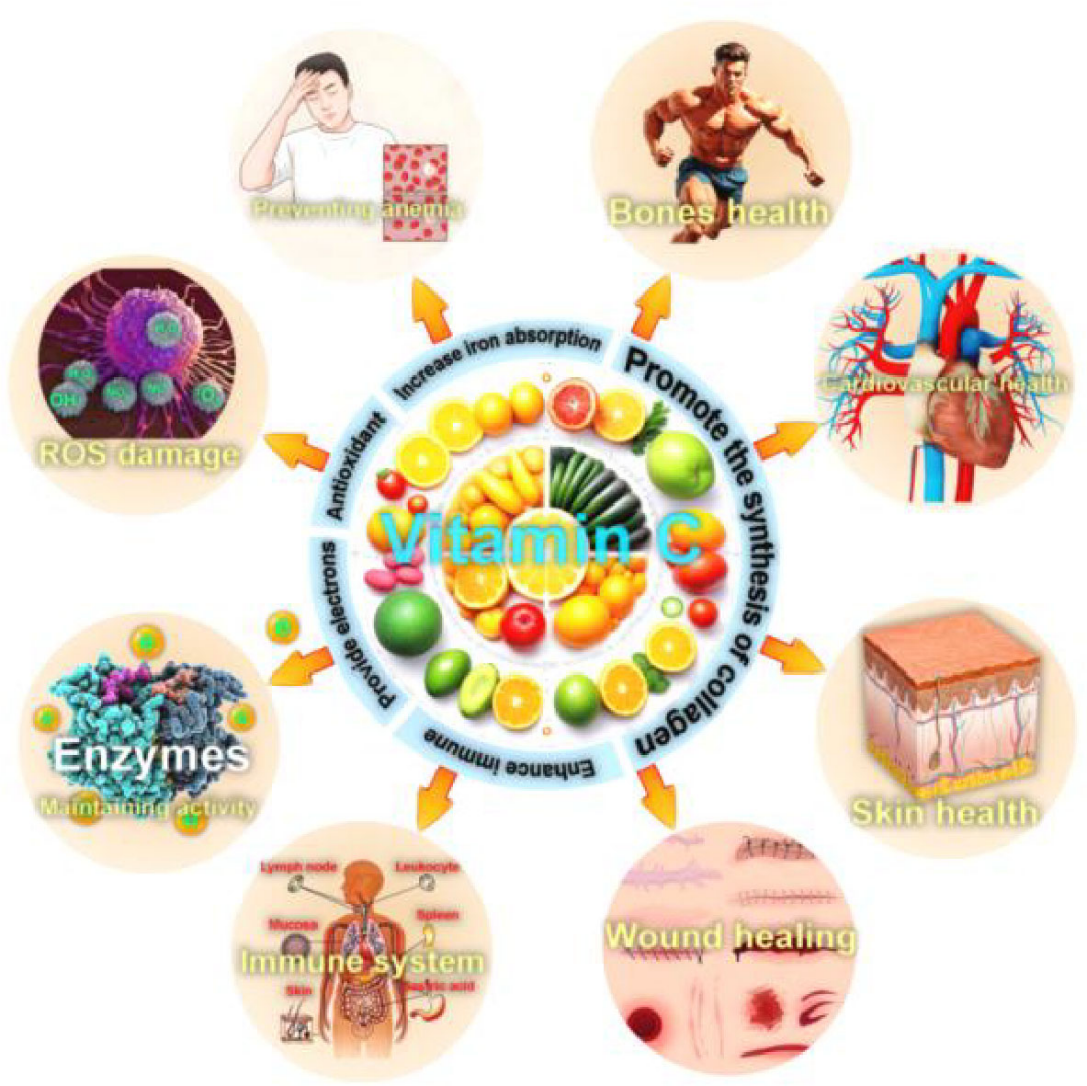
The source and functions of VC. the primary source of VC is fresh fruits and vegetables, including fruits such as oranges, grapefruits, and lemons, as well as strawberries, tomatoes, and various leafy green vegetables. the physiological concentration of VC plays several important roles in many critical metabolic reactions, including acting as an antioxidant to protect cells from ROS damage, promoting the synthesis of collagen to maintain the health of skin, bones, and blood vessels, increasing the absorption rate of iron to prevent anemia, enhancing the immune system’s function, promoting wound healing, maintaining cardiovascular health. VC, vitamin C; ROS, reactive oxygen species.

VC, a water-soluble polyhydroxy aromatic compound, exists in two enantiomeric forms—L-ascorbic acid (biologically active) and D-ascorbic acid (pharmacologically inert). Its redox instability in physiological environments (t_1/2_ < 2 h at pH 7.4) drives rapid oxidation to ascorbate radical (Asc^•–^) and dehydroascorbic acid (DHA), necessitating tight regulation of cellular redox buffering systems (24, 25). Humans, unlike most mammals, lack functional L-gulono-1,4-lactone oxidase (GULO), the terminal enzyme in VC biosynthesis, rendering dietary intake the sole source of this essential micronutrient (Figure 1) (26, 27). Dietary VC predominantly originates from citrus fruits (oranges, lemons), berries (strawberries, blackcurrants), and cruciferous vegetables (kale, broccoli), with intestinal absorption mediated by sodium-dependent VC transporters (SVCT1/SVCT2). At physiological concentrations (50–200 μM), VC serves dual roles as a master regulator of Figure 1 (28–33): (1) Metabolic homeostasis: Acts as an obligate cofactor for Fe^2+^-dependent dioxygenases, including collagen hydroxylases (proline/lysine modification) and HIF-1α prolyl hydroxylases (oxygen-sensing regulation). (2) Redox equilibrium: Scavenges ROS via two-electron donation, maintaining glutathione in its reduced state [glutathione (GSH)/oxidized glutathione (GSSG) ratio > 10:1]. (3) Epigenetic modulation: Facilitates TET enzyme-mediated DNA demethylation and Jumonji domain-containing histone demethylase activity, linking micronutrient status to chromatin remodeling. These functions collectively sustain critical physiological processes: collagen biosynthesis (triple-helix stabilization), iron absorption (ferric iron reduction in enterocytes), and immune cell activation (neutrophil chemotaxis enhancement). While urinary excretion of VC metabolites (oxalate, threonate) maintains systemic balance, prolonged deficiency (< 10 mg/day) disrupts connective tissue integrity, manifesting as scurvy, characterized by capillary fragility and impaired wound healing.

### 2.2 Biological distribution and metabolism

The physiological concentration of VC in human plasma ranges from 0.31 to 0.37 mM (34). The human body enters a relatively deficient state of VC after 72 h due to rapid utilization, decomposition rates and limited storage capacity. The concentrations of VC in the brain, adrenals, and leukocytes are relatively high and ranges from 1 to 15 mM, which is 15 to 200 times greater than that of plasma. The majority of cells *in vivo* facilitate the transfer of VC into the cytoplasm via the sodium-dependent VC transporters (*SVCTs*), including *SVCT1* and *SVCT2*. *SVCT1* is localized in the intestine and renal tubular epithelial cells and participates VC absorption and reabsorption (35). Different from *SVCT1*, *SVCT2* is the most abundant transporter distributed throughout the body and serves as the primary transporter of VC.

**FIGURE 2:**
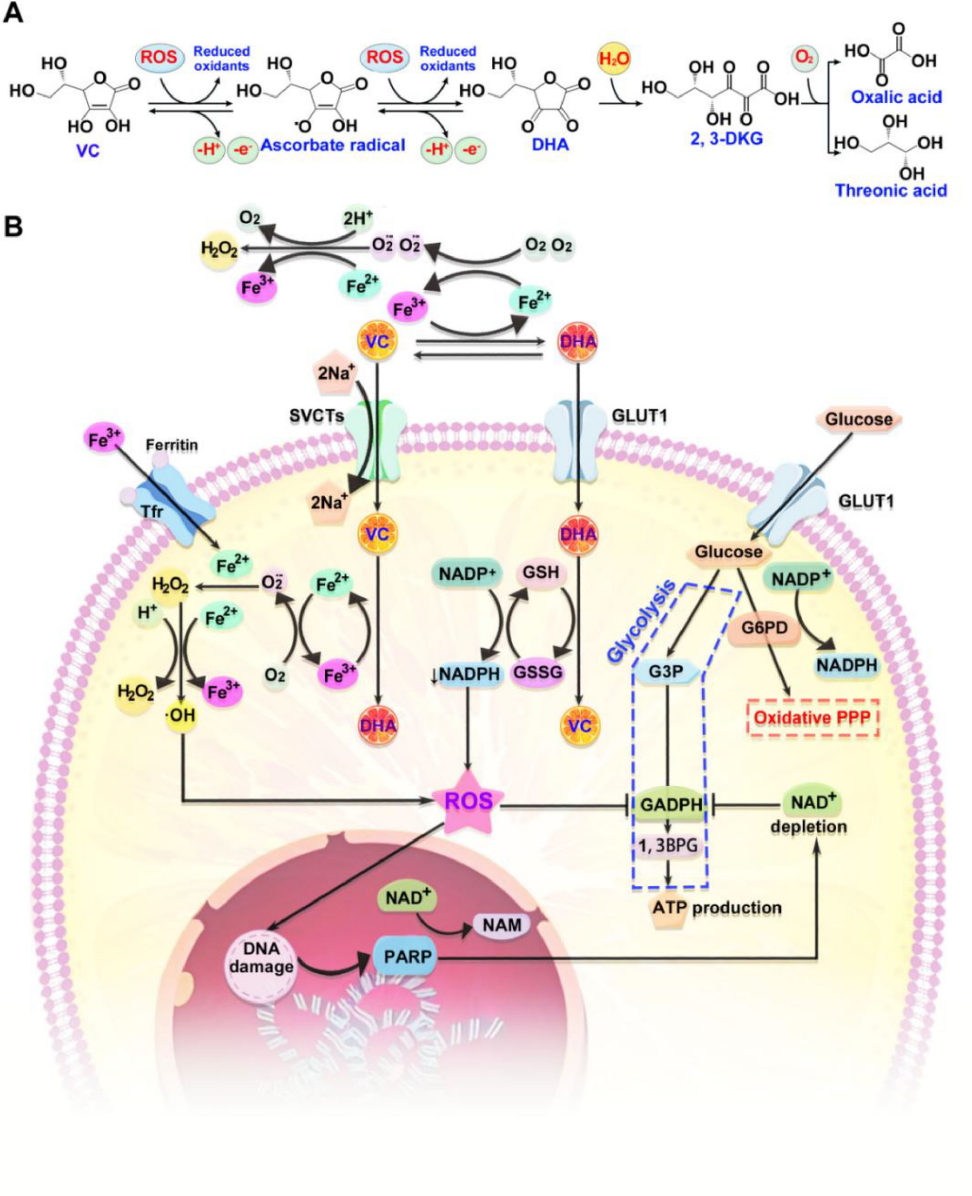
The metabolism of VC. **(A)** VC is susceptible to oxidation by ROS, resulting in the formation of ascorbate radical. This radical can subsequently be oxidized to *DHA*. Cells are capable of internalizing *DHA*, or it may undergo irreversible conversion to 2,3-DKG, which is ultimately metabolized into oxalic acid and threonic acid. **(B)** The intracellular metabolism of VC in cancer cells focuses on augmenting oxidative stress through two synergistic mechanisms. Firstly, extracellular H_2_O_2_ can directly induce cancer cell death by catalyzing the Fenton reaction to produce hydroxyl radicals (•OH). Secondly, H_2_O_2_ may elevate extracellular *DHA* levels by fostering a more oxidative tumor microenvironment. ROS, reactive oxygen species; DHA, dehydroascorbic acid; 2,3-DKG, 2, 3-L-Diketoglutonate; 1,3BPG, 1,3-bisphosphoglyceric acid; G3P, glyceraldehyde 3-phosphate; G6PD, glucose-6-phosphate dehydrogenase; GSSG, glutathione disulfide; PPP, pentose phosphate pathway; SVCTs, sodium-dependent VC transporters; NADPH, nicotinamide adenine dinucleotide phosphate.

The redox state of VC and its other forms (Asc^•–^ and *DHA*) is influenced by its biological milieu. *DHA* accounts for approximately 1–5% of the total VC content in the human body with short half-life of less than 1 min (36). A portion of *DHA* in the body is reduced back to VC by GSH, while the remaining *DHA* is irreversibly hydrolyzed to 2, 3-L-Diketoglutonate (*2,3-DKG*) and subsequently metabolized into oxalic acid and threonine (Figure 2A). *DHA* relies on glucose transporters (*GLUTs*) for its intracellular transportation (37). Among the more than 12 distinct *GLUTs*, *GLUT1* and *GLUT3* exhibit significantly greater affinities for *DHA* than for glucose. Under physiological conditions, the majority of *GLUTs* do not transport *DHA* due to the significantly higher plasma concentration for glucose (2–5 mM) than *DHA* (5–10 μM). Red blood cells and certain cancer cells do not express SVCTs and therefore rely on *GLUT1* or *GLUT3* for the transport of *DHA* to replenish VC (38). The resultant GSSG is subsequently regenerated to GSH through the utilization of *NADPH*. The continuously consumed intracellular reducing substances (such as GSH) by *DHA* accelerates its transportation by *GLUT1* or *GLUT3* with 10–20 times transport rate of that observed for SVCTs (39). Notably, the intracellular metabolism of VC in cancer cells focuses on augmenting oxidative stress through two synergistic mechanisms (Figure 2B). Firstly, extracellular H_2_O_2_ can directly induce cancer cell death by catalyzing the Fenton reaction to produce hydroxyl radicals (•OH). Elevated levels of labile ferric iron (Fe^3+^) in the tumor microenvironment promote the oxidation of VC, resulting in the formation of Asc^•–^, *DHA*, and ferrous iron (Fe^2+^). Subsequent oxidation of Fe^2+^ by oxygen yields superoxide anions (O_2_^•–^), which are converted to H_2_O_2_ and O_2_ by superoxide dismutase (SOD). Fe^3+^ can be internalized into cells via transferrin (*Tf*) binding to transferrin receptor (*TfR*), processed and oxidized in endosomes, contributing to the intracellular iron pool. The intracellular H_2_O_2_ reacts with labile Fe^2+^ to produce highly reactive hydroxyl radicals (•OH), detrimental to cellular integrity. The cycle is sustained by the reduction of Fe^3+^ to Fe^2+^ by VC and its radical, yielding fully oxidized VC, *DHA*. Secondly, H_2_O_2_ may elevate extracellular *DHA* levels by fostering a more oxidative tumor microenvironment. *DHA* can then enter cancer cells via *GLUT1*, depleting the intracellular reducing potential of reduced GSH and nicotinamide adenine dinucleotide phosphate (*NADPH*), thereby increasing intracellular ROS. This cascade activates poly (ADP-ribose) polymerase (*PARP*), a DNA repair enzyme, depleting cellular NAD^+^ levels, a necessary cofactor for *PARP*. NAD^+^ is also essential for glyceraldehyde 3-phosphate dehydrogenase (*GAPDH*) activity. Inhibition of *GAPDH* suppresses glycolysis in cancer cells, leading to diminished ATP production and subsequent cell death. This mechanism is potentiated by the frequent occurrence of high labile Fe^2+^ levels, *GLUT1* overexpression, and glycolytic dependency in cancer cells.

## 3 VC for cancer prevention

VC has shown potential in the prevention of certain types of cancer. As an antioxidant, it can neutralize free radicals and reduce oxidative stress, which may lower the risk of developing certain types of tumors (40). Additionally, VC plays a role in collagen synthesis, helping to maintain the integrity of cellular structures, and may enhance immune system function, thereby improving the body’s defense against tumor cells (41). The following text may focus on the administration, dosage, mechanisms, and applications of VC in cancer prevention.

### 3.1 Dosage and administration

**FIGURE 3:**
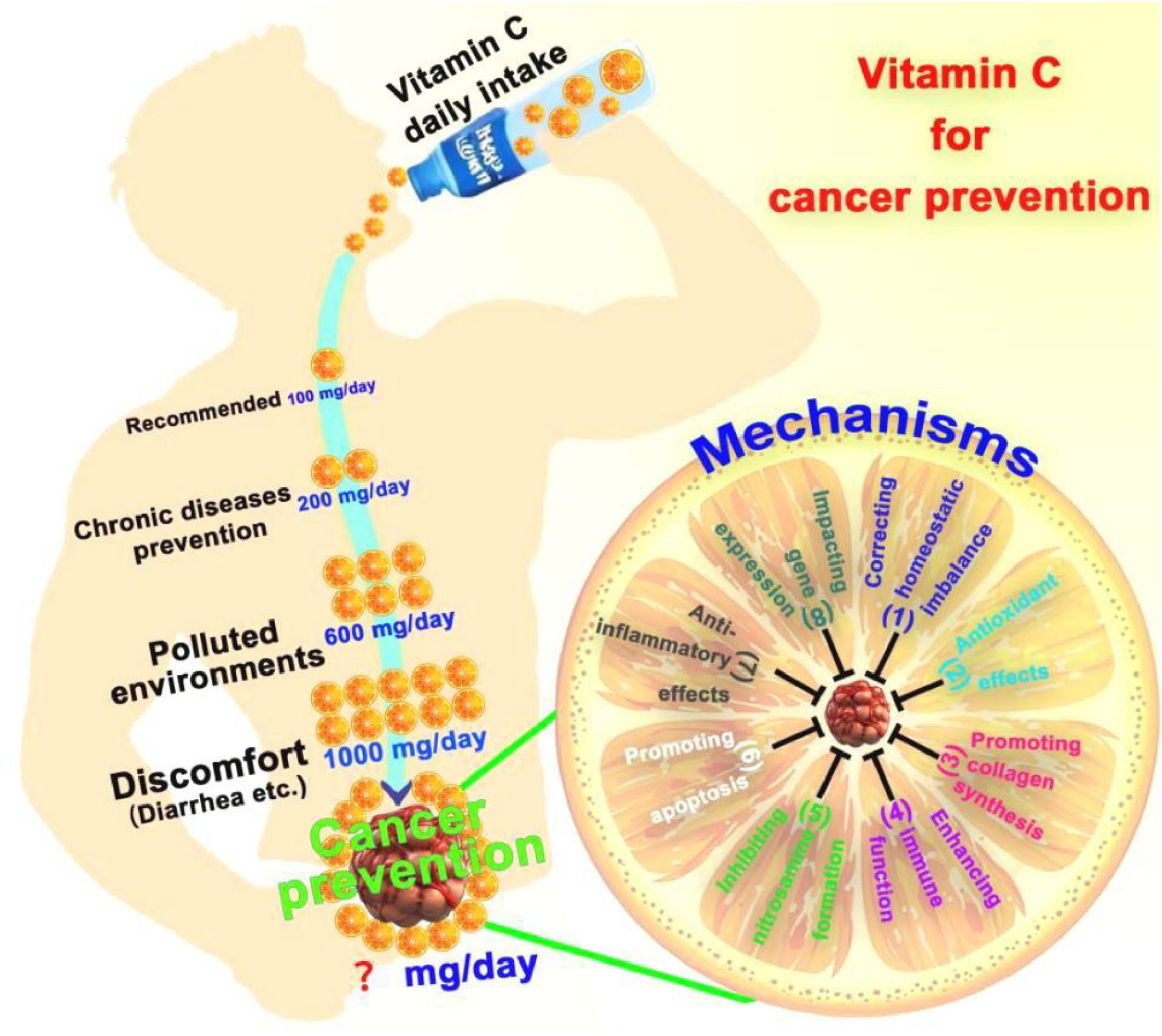
VC for cancer prevention. The optimal dosage of VC for cancer prevention is currently no established. Mechanisms of VC for cancer prevention include (1) correcting homeostatic imbalance, (2) antioxidant effects, (3) promoting collagen synthesis, (4) enhancing immune function, (5) inhibiting nitrosamine formation, (6) promoting apoptosis, (7) anti-inflammatory effects, (8) impacting gene expression.

The ingestion of VC-enriched foods or supplements by oral administration is a prevalent strategy for cancer prevention. Due to the constraints of gastrointestinal absorption and the swift urinary excretion, individuals often require high daily doses of VC to prevent cancer (41). Nonetheless, this approach results in suboptimal blood concentrations and a reduced duration of therapeutic effect (Figure 3). Given individual differences, there is currently no established gold standard for determining the optimal dosage of VC for cancer prevention. According to the Chinese Dietary Guidelines for Residents, the recommended daily intake of VC for Chinese residents is 100 mg, while it is 200 mg for the prevention of chronic diseases (42). Residents living in polluted environments should maintain a daily intake of VC of approximately 600 mg. Exceeding the maximum daily intake of 1,000 mg may lead to discomfort, such as diarrhea (43).

### 3.2 Mechanisms of VC for cancer prevention

Given the relatively low blood concentration of oral VC, its potential mechanism against cancer may be explained as follows (Figure 3): (1) Correcting the homeostatic imbalance. Addressing a series of homeostatic dysregulations associated with cancer development resulting from VC deficiency (44); (2) Antioxidant effects: VC is a potent antioxidant that can neutralize free radicals, reduce oxidative stress, thereby lowering the risk of DNA damage and cellular mutations, and preventing the onset of cancer (40); (3) Promotion of collagen synthesis: VC is involved in the synthesis of collagen, which helps maintain the integrity of the extracellular matrix between cells, thus preventing the spread and metastasis of cancer cells (45); (4) Enhancement of immune function: VC can enhance the function of the immune system, increase the activity and phagocytic capacity of white blood cells, and help the body more effectively identify and destroy cancer cells (41). (5) Inhibition of nitrosamine formation: VC can inhibit the formation of nitrosamines, which are strong carcinogens typically produced during food processing. VC reduces cancer risk by blocking their formation (46); (6) Influencing on cancer cell apoptosis: VC may promote apoptosis (programmed cell death) in cancer cells, thereby preventing tumor formation (47); (7) Anti-inflammatory effects: Chronic inflammation is associated with the development of various cancers, and VC has anti-inflammatory properties that can reduce inflammatory responses, thereby lowering cancer risk (48); (8) Impacting on gene expression: VC may affect the expression of specific genes, regulating cell proliferation and differentiation, and thus playing a role in the prevention of cancer (49).

### 3.3 Applications of VC for cancer prevention

Controversies exist regarding the cancer-preventive effects of VC. Agathocleous et al. (50) reported a negative correlation between the degree of hematopoietic stem cell differentiation and the VC content (Table 1). Specifically, a decrease in VC levels was associated with a significant increase in hematopoietic stem cell numbers, subsequently increasing the risk of leukemia development. Animal experiments demonstrated that knockout of the VC synthase GULO significantly promoted tumor development and shortened survival. The administration of VC prior to leukemia onset in mice effectively inhibited tumor formation. Restoring VC intake among leukemic mice extended their survival duration and improved treatment outcomes. Furthermore, oral ascorbic acid administered seven days prior to cancer cell inoculation reduced tumor progression in lymphoma xenograft models (51). Oral supplementation with VC was found to alleviate this condition Furthermore, similar conclusions were reached through clinical research analysis. Harris et al. (52) conducted a study involving 3,405 breast cancer patients and observed that those who received a substantial amount of VC supplementation prior to cancer diagnosis exhibited a 25% reduced risk of death compared to those who received minimal VC supplementation. A meta-analysis conducted in 2014 demonstrated that high-dose intake of VC significantly reduced the risk of lung cancer, with a 7% decrease in risk for every 100 mg/day increase in VC intake (53). A recent study employed Mendelian randomization to investigate the putative causal association between VC and the risk of digestive system cancer (54). The findings indicated that elevated levels of circulating VC are associated with reduced risks of small intestine and colorectal cancer, thereby implying a potential protective role for VC in the prevention of these malignancies.

**TABLE 1:** Applications of VC for cancer prevention.

Applications	Study type	Dosage and administration	Cancer type	Outcome	References
VC for cancer prevention	Animal	Oral	Leukemia	Extended survival duration and improved treatment outcomes (*P* < 0.05)	(50)
	Animal	Oral	Lymphoma	Reduced tumor progression	(51)
	Clinical	High-dose VC (∼1,000 mg), oral	Breast cancer	25% reduced risk of death (HR = 0.84; 95% CI = 0.71–1.00; *P* = 0.08)	(52)
	Meta-analysis	High-dose VC (every 100 mg/day increase in VC intake), oral	Lung cancer	High intake of VC against lung cancer (RR = 0.829, 95% CI = 0.734–0.937, I^2^ 5 = 57.8%); 7% decrease in risk for every 100 mg/day increase in VC intake (RR = 0.93, 95% CI = 0.88–0.98)	(53)
	Clinical	High-dose VC (500 mg/day), oral	Colon cancer	Lower risk of colon Cancer (OR = 0.84, 95% CI = 0.73–0.96; *P* = 0.013)	(54)

VC, vitamin C; HR, hazard ratio; CI, confidence intervals; RR, risk ratio; OR, odds ratio.

A randomized controlled clinical trial involving 14,641 men over 8 years of age who failed to demonstrated a reduction in prostate cancer or overall tumor risk with daily oral intake of 500 mg VC (55). Fu et al. (56) employed genotype data from 870,000 individuals and found no causal association between physiological concentrations of VC and the incidence of high-prevalence cancers, including lung, breast, prostate, and colorectal cancers. To further validate these findings, a prospective follow-up study encompassing approximately 2 million individuals and a meta-analysis of RCTs confirmed the absence of a significant association between oral VC intake and the risk of these high-risk cancers (57). However, the majority of VC anticancer research has been confined to basic *in vitro* experiments and small-scale clinical trials. High-quality randomized controlled clinical trials or multicenter, large-scale, prospective cohort studies remain scarce, necessitating further validation of the efficacy of supplemental VC in cancer prevention.

## 4 VC for cancer therapy

VC possesses potential clinical value in anti-cancer effects, possibly exerting its anti-tumor activity through mechanisms such as inhibiting tumor cell proliferation, inducing apoptosis, suppressing tumor angiogenesis, and metastasis. The specific anti-cancer mechanisms may involve antioxidant effects, inhibition of free radical damage, regulation of cell signaling pathways, enhancement of immune function, and promotion of collagen synthesis.

### 4.1 Dosage and administration

Both high concentrations of VC and *DHA* exhibit strong antitumor effects, while *DHA* is more toxic to cancer cells and has a higher transport capacity (58). However, it is less stable in neutral pH and has a shorter half-life than VC, making VC the preferred choice for anticancer therapy. When the concentration in plasma exceeds 1 mM, a range known as the pharmacological concentration, VC exhibits cytotoxic effects on the majority of tumor cells with respect to administration methods (59). Ou et al. (60) shown a direct correlation between fasting blood VC levels and tumor stage in patients with stage III–IV non-small cell lung cancer. As pathological staging advances, the VC blood concentration decreases and cannot even be detected for some patients, which provides crucial pathological evidence for VC supplementation in an anticancer manner.

**FIGURE 4:**
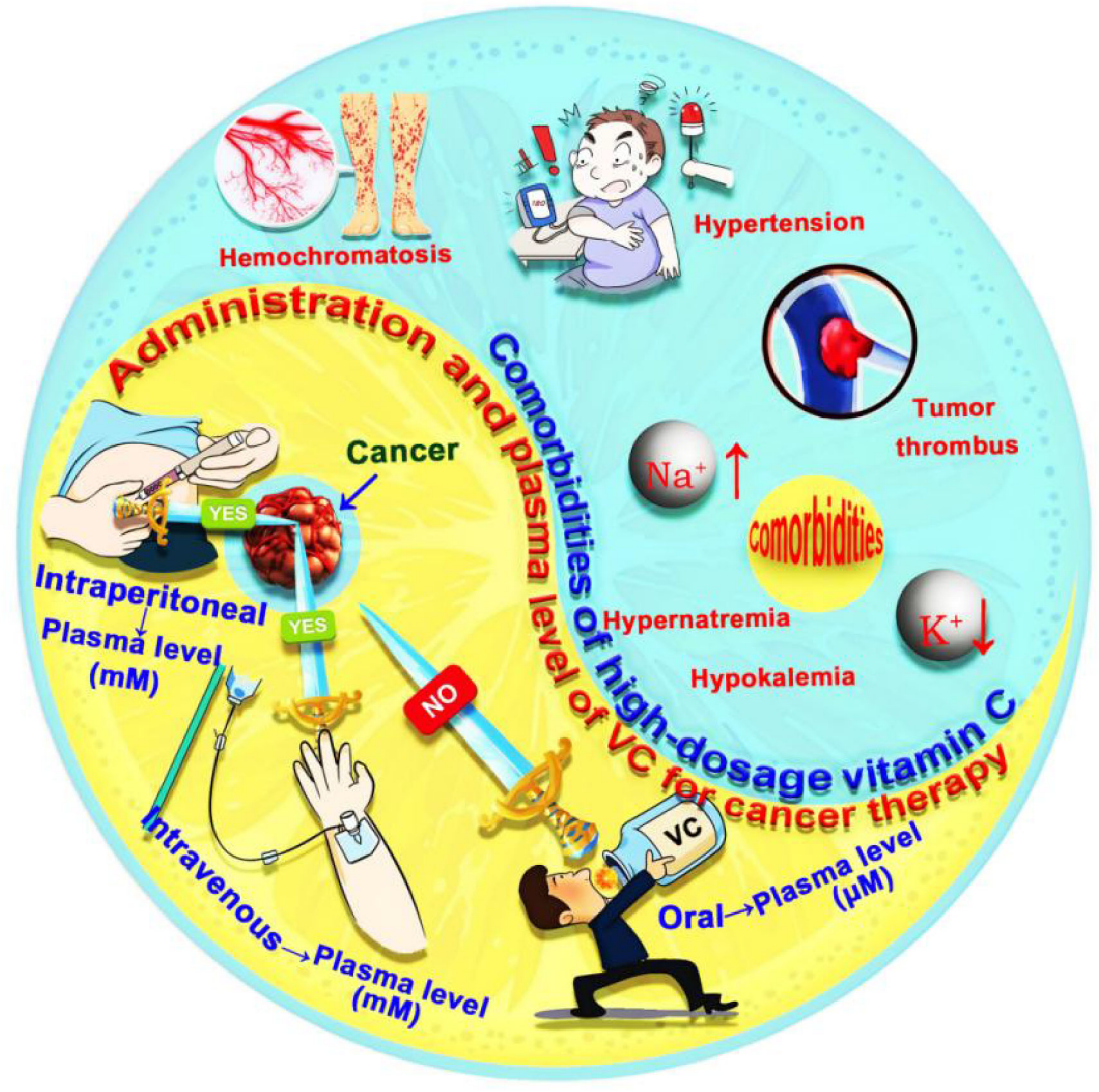
Administration methods and comorbidities of VC for cancer therapy. The administration methods VC for cancer therapy include oral, intravenous or intraperitoneal routes. The comorbidities of VC for cancer therapy include hypokalemia, hypernatremia, hypertension, iron overload in hemochromatosis, and tumor thrombus formation. VC, vitamin C.

The anticancer efficacy of high-dose VC is highly dependent on the method of administration, including oral, intravenous or intraperitoneal routes (Figure 4). Oral administration of 200 mg of VC results in a stable plasma concentration of about 80 μM. However, exceeding this dosage leads to a significant decrease in absorption and increased urinary excretion. Even with an oral regimen of 3 g of VC taken six times a day, peak plasma concentrations remain below 220 μM. Studies have shown that peak plasma concentrations from oral dosing do not exceed 0.3 mM, while *in vitro* research indicates that VC only demonstrates antitumor activity at concentrations above 0.5 mM (61–63). This suggests that oral administration may not achieve the necessary high concentrations for effective anticancer action *in vivo*. In contrast, intravenous or intraperitoneal administration allows for plasma concentrations that are 100 to 500 times higher than those from oral dosing (64). High-dose VC (1–4 g/kg) delivered via these routes can maintain plasma level above 10 mM for several hours, with peak concentrations reaching as high as 49 mM. Continuous daily dosing has been associated with tumor suppression rates of 40% to 60% and metastasis suppression rates of 50% to 90% (65, 66). Moreover, various proteomic, transcriptomic, and metabolomic studies have shown that intravenous administration of lower (100 μM) or medium (200 μM) doses can downregulate the cancer cell cycle and translational regulatory proteins, enhancing tumor suppression and elimination effects (67, 68). Thus, intravenous or intraperitoneal injection of high-dose VC, while maintaining blood concentrations within the pharmacological range, can effectively exert anticancer effects. Especially when VC is utilized solely for anticancer therapy, high dosages administered via intravenous injection are recommended. In cases where VC is combined with other anticancer treatments, the dosage can be appropriately reduced.

Clinical trials have demonstrated that patients receiving high-dosage intravenous VC therapy generally exhibit good tolerance. However, there is a risk of significant adverse effects due to non-specific organ accumulation, particularly in cancer patients with comorbidities such as hypokalemia, hypernatremia, hypertension, iron overload in hemochromatosis, and tumor thrombus formation (60, 63, 65) (Figure 4). Notably, It is imperative to maintain a vigilant stance regarding the long-term safety and potential toxicity associated with high-dose VC supplementation from following multiple aspects (30, 69–71). Chronic consumption of high-dose oral VC, particularly over 1 g daily, can increase the risk of kidney stones due to the production of oxalate, which may deposit in the urinary system and affect those prone to stones. Gastrointestinal side effects, such as diarrhea, stomach cramps, nausea, and vomiting may also occur with prolonged usage of high-dose oral VC. This is because excessive intake of VC, an acidic substance, can irritate the mucous membranes of the gastrointestinal tract, increase gastric acid secretion, and cause these uncomfortable symptoms. Long-term oral supplementation may also interfere with the absorption of essential minerals like copper and zinc. Furthermore, Special populations, such as diabetics, face additional risks. Diabetics should be cautious as HDIVC may potentially interfere with blood glucose monitoring. This interference can lead to falsely elevated glucometer readings on certain devices, due to the chemical reaction between VC and the glucose oxidase or glucose dehydrogenase enzymes utilized in some glucometers. To address these challenges, targeted delivery of VC to cancer cells through nanobioengineering technology has gained attention in research. This approach offers advantages such as high drug loading capacity, precise targeting, and controlled release. By enhancing the concentration of VC within target cells while minimizing adverse effects from non-specific accumulation, nanocarriers significantly improve the efficacy of VC-based cancer treatments (72, 73).

### 4.2 Anticancer mechanisms of VC

The anticancer mechanism of VC remains incompletely understood, yet it has been progressively revealed through further research. Currently, the anticancer mechanism of VC can be roughly divided into the following two aspects: cancer cell killing and tumor microenvironment remodeling.

#### 4.2.1 Regulating oncogene

**FIGURE 5:**
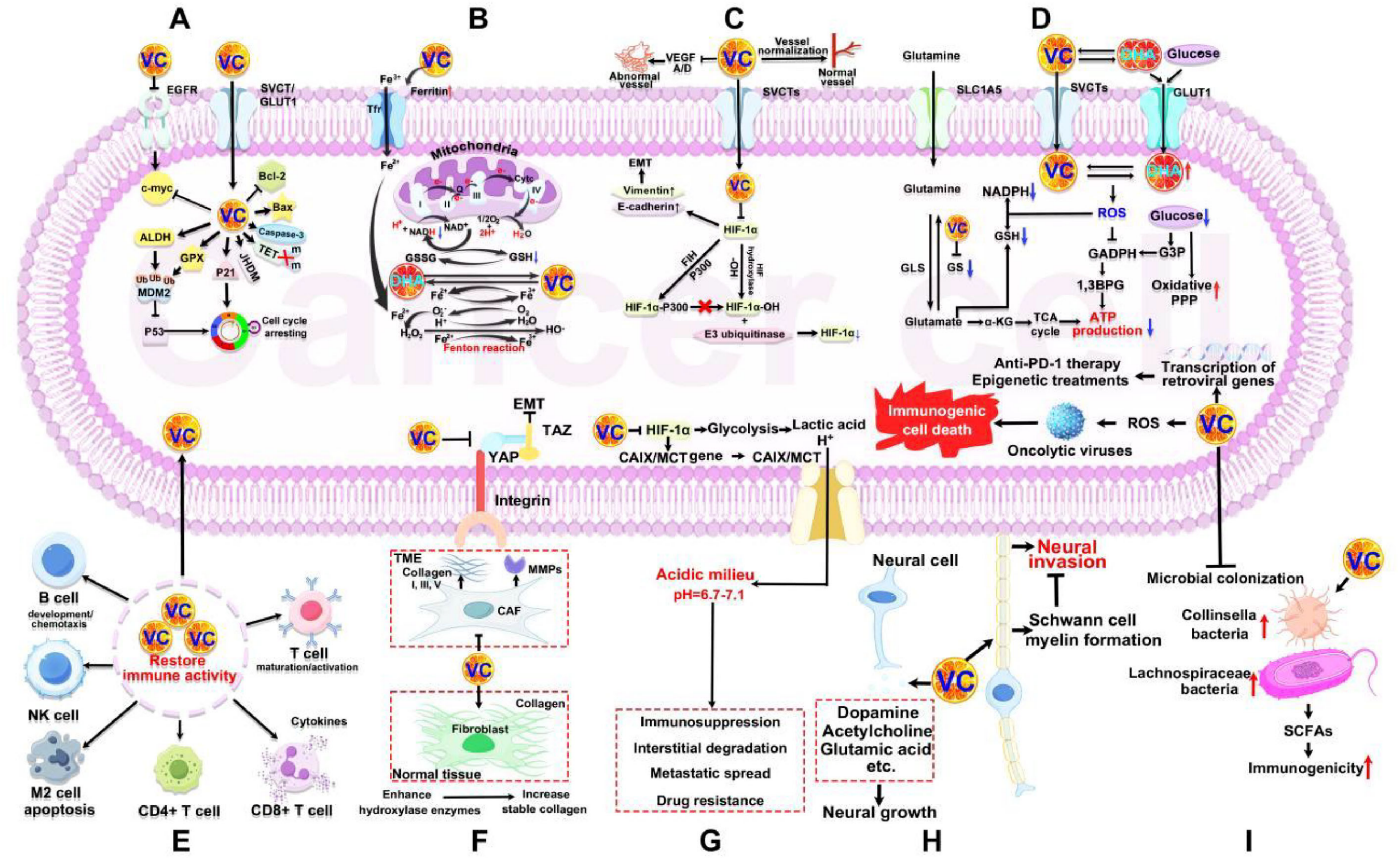
Anticancer mechanisms of VC. **(A)** Regulating oncogene. **(B)** Promoting oxidative stress. **(C)** Ameliorating hypoxic TME. **(D)** Inducing energy crisis. **(E)** Reversing immunosuppressive TME. **(F)** Remodeling extracellular matrix. **(G)** Reversing acidic TME. **(H)** Tumor neuromodulation. **(I)** Regulating microorganisms in TME. VC, vitamin C; EGFR, epidermal growth factor receptor; c-myc, cellular myelocytomatosis oncogene; SVCT, sodium-dependent VC transporter; GLUT1, glucose transporter 1; Bcl-2, B cell lymphoma 2; Bax, BCL-2-associated X protein; TET, 10–11 translocation; JHDM, Jumonji C domain (*JmjC*)-containing histone demethylase; GPX, glutathione peroxidase; ALDH, aldehyde dehydrogenase; MDM2, murine double minute 2; Tfr, transferrin receptor; NADH, nicotinamide adenine dinucleotide; GSH, glutathione; GSSG, oxidized glutathione; DHA, dehydroascorbic acid; EMT, epithelial-mesenchymal transition; FIH, factor-inhibiting HIF; HIF-α, hypoxia-inducible factor-1α; SLC1A5, recombinant solute carrier family 1, member 5; GS, glutamine synthetase; GLS, glutaminase; NADPH, nicotinamide adenine dinucleotide phosphate; ROS, reactive oxygen species; GADPH, glyceric acid phosphate dehydrogenase; G3P, glyceraldehyde 3-phosphate; 1,3BPG, 1,3-bisphosphoglyceric acid; TCA, tricarboxylic acid; ATP, adenosine triphosphate; NK cell, natural killer; TME, tumor microenvironment; YAP, yes-associated protein; TAZ, tafazzin; MMPs, matrix metalloproteinases; CAF, cancer-associated fibroblast; CAIX, carbonic anhydrase 9; MCT, monocarboxylate transporter; SCFAs, secretion of short-chain fatty acids.

VC plays a significant role in regulating gene expression, contributing to the prevention of malignant transformation and inhibiting the progression of tumors through various pathways (Figure 5A): (1) Downregulation of oncogenes. VC can downregulate the expression of the cellular myelocytomatosis oncogene (c-myc), which is associated with tumor growth and proliferation (74, 75). Dey et al. (76) found that VC inhibits the activation of epidermal growth factor receptor (*EGFR*) and its downstream target, c-myc, thereby reducing lung cell proliferation; (2) Activation of tumor suppressor genes. VC enhances the activities of glutathione peroxidase (*GPX*) and aldehyde dehydrogenase (*ALDH*) by transcriptionally activating the tumor suppressor gene p53. This activation is facilitated by the ubiquitination of murine double minute 2 (*MDM2*), which leads to the stabilization and activation of p53, promoting programmed cell death in cancer cells (77); (3) Cell cycle arrest. VC can activate the cyclin-dependent kinase inhibitor p21 and upregulates p53, leading to cell cycle arrest at the G0/G1 phase and thus limiting their proliferation (78); (4) Modulation of apoptotic factors. High doses of VC can downregulate the expression of the anti-apoptotic gene *Bcl-2* and upregulate pro-apoptotic factors such as Bax and cleaved caspase-3 (79). This shift in the balance between pro- and anti-apoptotic factors ultimately leads to increased apoptosis in tumor cells.

Cancer cells often display significant DNA and RNA methylation due to elevated levels of DNA methyltransferases (DNMTs) and reduced activity of the TET proteins responsible for demethylation. VC can enhance the residual demethylation activity of TET proteins in cancer cells, leading to the re-expression of tumor suppressor genes, thereby interfering with tumor survival and enhancing sensitivity to other cancer treatments (80). Preclinical studies have indicated that high-dose VC can block the abnormal proliferation of hematopoietic stem cells by activating cellular responses to changes in energy metabolism, oxygen, and iron levels (81, 82). This occurs through the restoration activity of TET and the enhancement of Jumonji C domain (JmjC)-containing histone demethylase (JHDM) (Figure 5A).

#### 4.2.2 Promoting oxidative stress

Cancer cells often exhibit mitochondrial dysfunction, an unstable intracellular iron pool, low catalase activity, and inadequate ROS scavenging capabilities. High concentrations of VC can induce cancer cell death through prooxidant effects for several reasons (Figure 5B): (1) Impact on mitochondrial function. High concentrations of VC can influence cytochrome C and the nicotinamide adenine dinucleotide (NADH) ubiquinone reductase complex I within the mitochondrial electron transport chain (83–85). This effect is mediated by altering Fe^2+^-dependent dioxygenase activity in cancer cells, which upregulates the endoplasmic reticulum unfolded protein response and proteins related to translation inhibition, such as eukaryotic initiation factor 2α (eIF2α) and protein kinase R (PKR)/PKR pTr-446. This leads to increased mitochondrial and endoplasmic reticulum (ER) stress responses; (2) Iron metabolism alterations. VC can promote the synthesis of ferritin and inhibit its degradation by inhibit nuclear factor kappa-B (NF-κB) in cancer cell, resulting in an increase in Fe^3+^ within the cells and its subsequent reduction to Fe^2+^. The elevated unstable iron pool exacerbates the Fenton reaction, generating more ROS and heightening intracellular oxidative stress (8). The ROS produced can inhibit the phosphorylation of inhibitor of nuclear factor-κB kinase (IKK) α/β and inhibitory subunit of NF kappa B alpha (IκBα), creating a positive feedback loop that blocks the activation of the IKK/IκB/NF-κB pathway (86). Additionally, DHA, as the oxidized form of VC, can be reduced back to VC by consuming GSH within the cell, further amplifying oxidative stress levels. Excessive ROS in cancer cells can inhibit and kill these cells through several mechanisms (87, 88) (Figure 5B): (1) Cellular damage. High levels of ROS can damage DNA, proteins, and lipids, leading to oxidative stress in the endoplasmic reticulum and mitochondria of cancer cells; (2) Inhibition of kinase activity. ROS can inhibit the activity of key intracellular kinases, including mitogen-activated protein kinase (MAPK)/extracellular regulated protein kinases (ERK), phosphatidylinositol 3-kinase (PI3K)/protein kinase B (AKT), and BRAF, which are crucial for cell survival and proliferation; (3) Cysteine modification. ROS can oxidize and modify cysteine-containing proteins during translation and the cell cycle, resulting in the arrest of cancer cells in the S phase. This is especially relevant in tumors that rely on antioxidants to maintain their intracellular redox balance. Ma et al. (89) demonstrated that high doses of VC administered via tail vein injection in glioma model mice led to increased activity of checkpoint kinase 2 (CHK2) and histone 2AX (H2AX), indicating that high-dose VC caused DNA damage by elevating intracellular ROS levels. Moreover, VC can catalyze the reaction between superoxide anions and Fe^2+^ to produce hydrogen peroxide outside the cell, contributing to cancer cell death. Notably, high-dose VC appears to spare normal cells *in vivo* from damage while exerting its anticancer effects. This difference is primarily due to the lower unstable iron pool and stronger ROS scavenging capabilities found in normal cells compared to cancer cells. The above selective toxicity underscores the potential of VC as a therapeutic agent in cancer treatment.

#### 4.2.3 Ameliorating hypoxic TME

In solid tumors, the mismatch between blood vessel formation and tumor growth often leads to hypoxia, as tumor cells obstruct surrounding blood vessels and inhibit new capillary formation (90) (Figure 5C). The hypoxic microenvironment inhibits the hydroxylation of proline residues on HIF-1α by proline hydroxylase (PHD), which prevents HIF-1α from binding to the tumor suppressor protein VHL, thereby avoiding its degradation by E3 ubiquitinase. Simultaneously, hypoxia also impairs the hydroxylation of asparagine residues on HIF-1α by factor-inhibiting HIF (FIH), facilitating its binding to p300. These processes collectively enhance the transcriptional activity of HIF-1α, activating various genes that promote tumor survival. Importantly, HIF hydroxylase, an α-KG-dependent dioxygenase, requires VC as a cofactor for iron cycling (91). Therefore, VC supplement can enhance HIF hydroxylase function, thereby reducing HIF-1α expression and inhibiting tumor progression. For example, previous study showed a significant negative correlation between intratumoral HIF-1α levels and VC in endometrial, renal, and colon cancers, with higher VC levels associated with better postoperative outcomes (92). The potential mechanism could be ascribed to high-dose VC decreasing HIF-1α and its downstream targets (GLUT-1 and VEGF). Zhao et al. (93) and Zeng et al. (94) fund that high-dose VC can decrease vimentin levels and increase E-cadherin expression, effectively reversing the epithelial–mesenchymal transition (EMT) in tumor cells. This reversal is achieved by VC inactivating the HIF-1α, which inhibits cell migration and invasion, thereby potentially limiting tumor progression and metastasis. Tumor neovascularization is marked by high permeability and discontinuity, which can facilitate distant metastasis while also obstructing immune cell infiltration and drug delivery. VC has been shown to downregulate the expression of VEGF-A and VEGF-D, thereby inhibiting abnormal angiogenesis (95, 96). Additionally, VC promotes vessel normalization and reduces vascular permeability by inducing endothelial cell contraction and preventing endothelial cell apoptosis. These multifaceted actions of VC may enhance the efficacy of therapies targeting tumors by improving drug delivery and immune response.

#### 4.2.4 Inducing energy crisis

Cancer cells frequently exhibit a dependency on aerobic glycolysis, also known as the Warburg effect, and glutamine addiction to procure glucose and glutamine from their microenvironment for energy feeding (97) (Figure 5D). VC has been shown to impede these energy acquisition pathways through multiple mechanisms. It induces elevated levels of ROS within cancer cells, which in turn suppresses glycolysis by inhibiting GAPDH activity. VC promotes a metabolic shift in glutamine utilization toward the synthesis of GSH, thereby replenishing intracellular GSH levels and competitively inhibiting glutamine-dependent energy production. VC facilitates the degradation of glutamine synthetase, which diminishes the endogenous supply of glutamine for metabolic energy production. These alterations compel cancer cells to rely on the more energetically demanding oxidative phosphorylation pathway for energy acquisition. As described in section “2.1 Biological properties and functions,” it was established that cancer cells expressing GLUT1 transport DHA at a faster rate than glucose, and that the ROS released by cancer cells in response to DHA can further enhance its uptake. Consequently, for tumors with KRAS or BRAF mutations (such as colon and pancreatic cancers) that exhibit high GLUT1 expression and low SVCTs expression, extracellular DHA treatment competitively inhibits glucose uptake by cancer cells. This impairment of glycolysis consequently reduces the cells’ energy production. Simultaneously, the treatment creates a significant concentration gradient of DHA across the cancer cell membrane. The accelerated influx of DHA into cancer cells leads to the generation of copious amounts of ROS. The production of ROS depletes intracellular NADPH and disrupts the GAPDH-mediated glycolytic flux between glyceraldehyde 3-phosphate (GAP) and 1,3-bisphosphoglyceric acid (1,3-BPG). Furthermore, the elevated ROS levels increase pentose phosphate pathway (PPP) enzyme activity while diminishing the tricarboxylic acid (TCA) cycle and downstream glycolytic products (98). These combined effects result in decreased intracellular ATP production, culminating in an energy crisis and the demise of the cancer cells.

#### 4.2.5 Reversing immunosuppressive TME

Most immune cells rely on high intracellular concentrations of VC to exert antioxidant effects, maintain redox balance, and provide electrons necessary for the activity of iron and copper-dependent dioxygenases, which are crucial for normal immune function (Figure 5E). However, the uptake of VC from TME by cancer cells can lead to a deficiency in immune cells, resulting in immune dysfunction. Therefore, supplementing with VC can help restore the antitumor immune activity of these cells. This includes promoting the differentiation and polarization of myeloid and T cells, enhancing T cell maturation and activation, increasing T cell infiltration, and improving the CD8+/CD4+ T cell ratio (99, 100). VC supplementation can also support the development and chemotaxis of B cells, boosts cytokine production, induces apoptosis in M2-type macrophages, and enhances natural killer (NK) cell-mediated tumor killing (101–103).

#### 4.2.6 Remodeling extracellular matrix

Cancer-associated fibroblasts (CAFs) play a crucial role in extracellular matrix (ECM) remodeling by producing type I, III, and V collagen, as well as fibronectin, and secreting matrix metalloproteinases (MMPs) (104) (Figure 5F). This remodeling contributes to tumor tissue stiffness and provides a supportive scaffold for tumor cell survival, proliferation, and metastasis. During the early stages of metastasis, cancer cells can detect changes in the mechanical properties of the ECM, transmitting mechanical signals that trigger cytoskeletal remodeling and EMT. VC is pivotal in the above processes. In terms of ECM formation, VC significantly downregulates the expression of genes associated with collagen synthesis, cell adhesion, and the ECM in CAFs (105, 106). As a cofactor for hydroxylase enzymes, VC enhances proline and lysine hydroxylation, increasing collagen content in normal tissue and promoting the formation of a stable three-dimensional collagen structure, which helps prevent cancer cell invasion (107). VC can inhibit ECM remodeling through several mechanisms: (1) Reducing the mRNA expression of various MMPs in CAFs, thereby blocking ECM remodeling and the secretion of tumor-promoting growth factors (105); (2) Inhibiting tumor cell EMT by modulating integrin and YAP/TAZ mechanical signaling (108); (3) Enhancing proline hydroxylation and further promoting collagen-integrin adhesion and ERK1/2 phosphorylation in cell stretching pathways (109).

#### 4.2.7 Reversing acidic TME

Long-term high rates of aerobic glycolysis often leads to the accumulation of acidic substances (such as lactic acid and H^+^) in TME, resulting in an acidic milieu with a pH of 6.7 to 7.1 (110) (Figure 5G). This acidic TME adversely affects various aspects of malignant tumors, including immunosuppression, interstitial degradation, metastatic spread, and drug resistance. VC, known as a glycolysis inhibitor, can directly inhibit HIF-α-mediated glycolysis and the expression of downstream genes associated with acidic metabolites in both cancer cells and stromal cells within the microenvironment. This action helps reduce the levels of endogenous acidic metabolites. Additionally, VC downregulates the gene expression of hypoxia-induced carbonic anhydrase 9 (CAIX) and monocarboxylate transporter (MCT), thereby decreasing H^+^ generation and inhibiting the efflux of H^+^ and lactic acid (111, 112).

#### 4.2.8 Tumor neuromodulation

In addition to neurons, glial cells, such as Schwann cells and oligodendrocytes, play a key role in neuro-tumor communication. Zhang et al. (113) found that Schwann cells promote the neural invasion of cancer cells through autophagy based on a study of pancreatic cancer. Englard and Seifter (114) reported that VC is abundant in neurons and can be used as a neuromodulator to promote the biosynthesis of norepinephrine in nerve cells by enhancing the activity of dopamine β hydroxylase (Figure 5H). Huff et al. (115) demonstrated that VC can boost proline hydroxylase activity in Schwann cells, promoting collagen synthesis and inducing demyelination of Schwann cell myelin genes to enhance myelin formation. Some studies have also shown that VC can promote the storage and release of neurotransmitters (dopamine, acetylcholine, glutamic acid, etc.), promote neural growth and development, and mediate the metabolic coupling between glial cells and neurons through the ascorbic acid cycle (116). Conversely, Ferrada et al. (117) found that a physiological dose of VC (200 μM) could induce necrotic apoptosis in neurons *in vitro*. While VC plays a role in maintaining neurobiological function within the tumor microenvironment, strong evidence supporting its regulatory effects on tumor-associated nerves is still lacking, and further exploration of the related functions and mechanisms is needed.

#### 4.2.9 Regulating microorganisms in TME

The immune-tumor-microorganism axis has emerged as a key focus in tumor diagnosis and treatment research. The microbial TME includes intestinal and intratumoral microorganisms and their metabolites, which significantly influence tumor development (118). VC can modulate tumor progression by regulating microbial balance both in the intestine and within tumors. Some studies indicated that oral VC administration increases the abundance of Collinsella and Lachnospiraceae bacteria in feces, leading to heightened secretion of short-chain fatty acids (SCFAs) that enhance the immunogenicity of various cancers (119, 120) (Figure 5I). This suggests a novel role for VC in improving cancer immunotherapy. Notably, VC can also strengthen the tumor’s physical barrier, inhibit microbial colonization, and promote the transcription of retroviral genes in tumor cells, thereby enhancing the effectiveness of anti-PD-1 immune checkpoint therapy and epigenetic treatments (103). Moreover, the research by Ma et al. (121) demonstrated that ROS generated by high-dose VC can induce immunogenic cell death mediated by oncolytic viruses, transforming the tumor into a “hot” environment conducive to immune response.

### 4.3 Therapeutic applications of VC

High-dose VC has demonstrated potent anticancer effects by disrupting cancer cell homeostasis and inhibiting tumor proliferation, invasion, and metastasis. For instance, Ryszawy et al. (122) found that high-dose VC induced excessive ROS production and necrosis in glioblastoma and pancreatic cancer cells, leading to significant reductions in cell proliferation and motility. Multiple clinical studies have also shown that long-term VC use in female patients with breast cancer significantly reduces recurrence risk and disease-related mortality (123–126). Lamm et al. (127) reported that in a study of 65 bladder cancer patients, those receiving high-dose VC alongside Bacillus Calmette-Guérin (BCG) immunotherapy experienced a notable decrease in tumor recurrence after 10 months. Overall, VC exhibits anticancer properties both as a standalone treatment and in combination with other therapies. The following section may summarize the latest applications of VC in cancer treatment (Table 2).

**TABLE 2:** Applications of VC for cancer therapy.

Applications	Study type	Dosage and administration	Cancer type	Outcome	References
VC as a monotherapy for cancer	Cell line	High-dose VC (1, 5, 10, 15, and 20 mM)	Breast cancer	Induced dose-dependent tumor spheroid cell death (*P* < 0.05).	(128)
	Cell line	High-dose VC [100 μg/ml (0.345 mM)]	IDH1 mutant acute myeloid leukemia	Induced differentially methylated regions that displayed a significant overlap with enhancers implicated in myeloid differentiation (*P* < 0.05).	(129)
	Cell line	High-dose VC (≥ 20 mM)	Triple-negative breast cancer	Intrinsic apoptosis (*P* < 0.05)	(130)
	Animal	High-dose VC (4 g/kg), intravenous	Acute myeloid leukemia	Leukemia progression by inhibiting hematopoietic stem cell proliferation and reducing leukocyte counts (*P* < 0.05).	(131)
	Animal	High-dose VC (3.3 g/kg), intravenous	Osteosarcoma	induced non-apoptotic cancer cell death in osteosarcoma models (*P* < 0.05).	(132)
	Animal	High-dose VC (4 g/kg), intraperitoneal	*KRAS* and BRAF mutant colorectal cancer	Stronger anticancer effect in animals with *KRAS* and BRAF mutant by decreasing intracellular glucose and energy production due to intracellular ROS accumulation and GAPDH inhibition compared wild type tumor (*P* < 0.05).	(133)
	Animal	High-dose VC (4 gr/Kg), intraperitoneal	*KRAS* mutant colon cancer	Inhibited colon cancer growth (*P* < 0.05).	(134)
	Animal	High-dose VC (4 g/kg), intravenous	Glioblastoma multiforme, non-small-cell lung cancer	Retardation of tumor growth and invasion (*P* < 0.05).	(135)
VC enhancing single anticancer therapy	Cell line	High-dose VC (250 μM), intravenous	Acute myeloid leukemia	VC in combination with PARP inhibition enhances cell death (*P* < 0.05).	(131)
	Animal	High-dose VC (4 g/kg per day), intra peritoneal	Melanoma, colorectal, pancreatic, and breast cancer	Antiproliferative effect; delays tumor growth (*P* < 0.05).	(100)
	Animal	High-dose VC (1.5 M/150–200 μL), intra peritoneal	Lymphoma	High-dose VC treatment synergizes with anti-PD1 checkpoint inhibition resulted in significant tumor Proliferation inhibition (*P* < 0.05).	(103)
	Animal	High-dose VC (0.5 g/kg), intra peritoneal	*KRAS* mutant colon cancer	Enhancing anti-*EGFR* efficacy of cetuximab by increasing ROS production in *KRAS* mutant colon cancer cell xenografts overexpressing *SVCT2* (*P* < 0.05).	(141)
	Clinical	High-dose VC (4 g/kg), intravenous	Ovarian cancer	Mitigated adverse reactions and enhanced therapeutic efficacy (*P* < 0.05).	(142)
	Clinical	High-dose VC (15 g/250 mL, 600 mg/m^2^ over 30 min), intravenous	Pancreatic cancer	Improving median overall survival (VC group: 21.7 months vs. control group: 12.7 months, *P* = 0.08) and progression-free survival (VC group: 13.7 months vs. control group: 4.6 months, *P* < 0.05).	(143)
	Clinical	High-dose VC (1 g/kg), intravenous	Non-small cell lung cancer	Significantly improved the quality of life and prolong the survival time [VC plus best supportive care (BSC) group vs. BSC group: progression-free survival (PFS) (3 months vs. 1.85 months, *P* < 0.05); overall survival (OS) (9.4 months vs. 5.6 months, *P* < 0.05)]	(144)
VC enhancing multiple anticancer therapies	Cell line	Pharmacological doses of VC (100 to 250 μM)	Breast cancer	Synthetic lethal strategy for eradicating cancer stem cells (*P* < 0.05).	(145)
	Cell line	Daily dose of 57 μM	Acute myeloid leukemia, breast carcinoma, and hepatocellular carcinoma	Improving responses to epigenetic therapy with DNA methyltransferase inhibitors (*P* < 0.05).	(146)
	Animal	High-dose VC (4 g/kg), intra peritoneal	*KRAS* mutant colorectal cancer	Reducing tumor growth and extending mouse survival (*P* < 0.05).	(147)
	Clinical	High-dose VC (1 g/kg), intravenous	Triple-negative breast cancer cells	Significantly prolonged the progression-free survival and overall survival of patients (*P* < 0.05).	(148)
	Clinical	High-dose VC (1.5 g/kg), intravenous	*KRAS* mutant colon cancer	Longer PFS in patients receiving HDIVC plus FOLFOX ± bevacizumab group rather than FOLFOX ± bevacizumab alone (9.2 vs. 7.8 months, HR 0.67; 95% CI, 0.50–0.91; *P* = 0.01)	(149)
VC alleviating the side effects of cancer treatment	Clinical	High-dose VC (200 mg/kg), oral	Breast cancer metastasis	Inhibiting effectively the adverse effect of radio therapy or chemotherapy, such as, severe nausea, or frequent diarrhea in cancer therapies.	(150)
	Clinical	High-dose VC (7.5 g–50 g), intravenous	Multiple-type cancers	Reducing inflammation in cancer patients (*P* < 0.05).	(151)
	Clinical	10 g VC twice with a 3-day interval, intravenous	Multiple-type cancers	Health score improved from 36 ± 18 to 55 ± 16 after administration of VC (*P* = 0.001).	(152)

VC, vitamin C; IDH1, isocitrate dehydrogenase 1; KRAS, kirsten rat sarcoma viral oncogene; BRAF, B-Raf proto-oncogene.

#### 4.3.1 VC as a monotherapy for cancer

High-dose VC has been extensively studied as a potential monotherapy for various cancers, including hematological malignancies like leukemia, as well as solid tumors such as colorectal, pancreatic, and lung cancers. Mussa et al. (128) reported that VC induced dose-dependent tumor spheroid cell death, primarily due to elevated H_2_O_2_ production and oxidative stress imbalance caused by GSH depletion, with varying susceptibilities between MCF-7 and MDA-MB-231 spheroids. VC also showed efficacy against acute myeloid leukemia with IDH mutations, a common genetic alteration associated with poor prognosis (129). Furthermore, studies on triple-negative breast cancer stem cell lines (MDA-MB-231 and MDA-MB-468) showed differential but excellent therapeutic responses to VC, suggesting its potential as a targeted treatment strategy (130). Additionally, research by Cimmino et al. (131) demonstrated that daily high-dose VC injections in mice with low TET2 activity slowed leukemia progression by inhibiting hematopoietic stem cell proliferation and reducing leukocyte counts. Beyond hematologic cancers, VC has also shown promise in solid tumors. Vaishampayan and Lee (132) demonstrated that pharmacological VC induced non-apoptotic cancer cell death in osteosarcoma models via an intracellular ROS-iron-calcium crosstalk mechanism, leading to mitochondrial dysfunction and reduced ATP levels, with potential clinical utility in osteosarcoma treatment. A study published in Science in 2015 revealed that high-dose VC could trigger apoptosis of BRAF or KRAS mutant colon cancer cells by decreasing intracellular glucose and energy production due to intracellular ROS accumulation and GAPDH inhibition, which was not observed in wild type colon cancer cells (133). In genetically engineered mice with KRAS-driven colon tumors, VC treatment led to fewer and smaller tumors. Cenigaonandia-Campillo et al. (134) found that higher doses of VC (5–10 mM) inhibited KRAS mutant colon cancer growth in both *in vivo* and *in vitro* studies by reducing mitochondrial membrane potential and levels of ATP and GLUT1 in cancer cells. Previous research for brain and lung cancers have confirmed the safety of high-dose VC, showing alterations in cellular iron metabolism and increased reactive oxygen species, which contribute to cancer cell death (135). Clinical phase II studies are currently evaluating the effectiveness of high-dose VC monotherapy in metastatic colorectal, pancreatic, and lung cancers, particularly those with KRAS or BRAF mutations (136) (Table 3). Other clinical studies of VC monotherapy for cancer that have explored the antitumor effect of low-to-moderate doses or oral VC monotherapy (137–139). However, as mentioned earlier, most basic research has shown that low and medium doses of VC or oral VC have no anticancer effect. Only HDIVC can exert the independent anticancer effect of VC. Nevertheless, VC’s impact may not be as robust as first-line treatments like chemotherapy or radiotherapy, indicating its potential role as an adjuvant therapy to enhance the efficacy of existing cancer treatments. As RAF family protein kinases are a key node in the RAS/RAF pathways in the signaling cascade, KRAS mutations typically cause broad disruption to RAS pathway signaling (140). This includes impairment of the antioxidant response and a heightened reliance on glycolysis. In contrast, BRAF mutations often allow compensatory mechanisms within the pathway to remain functional, such as mitochondrial metabolism. Consequently, compared to BRAF-mutant tumors, KRAS-mutant tumors may demonstrate elevated vulnerability to oxidative stress and epigenetic modulation triggered by high-dose VC. However, the differential sensitivity of KRAS- versus BRAF-mutated tumors to high-dose VC remains unexplored empirically and thus warrants further investigation.

**TABLE 3:** The onging clinical trials of VC for cancer therapy.

ID	Phase	Dosage and administration	Cancer type	Primary outcome	References
NCT03146962	II	1.25 g/kg, intravenous.	Metastatic colorectal, pancreatic, and lung cancer	Pathologic response based on tumor regression; 3-month DCR; maximal tolerated dose of high-dose VC.	(136)
NCT04046094	I/II	25 g infused 2 times a week for 4 weeks, intravenous.	Cisplatin-ineligible, muscle invasive bladder cancer	Post treatment pathological staging.	(137)
NCT03682029	Not Applicable	500 mg/capsule. Ingestion of 2 capsules (1,000 mg) daily, oral	Low-risk myeloid malignancies	Median change from baseline in variant allele frequency at 12 months.	(138)
NCT03613727	II	50 mg/kg/day, Intravenous; After completion of intravenous VC, oral VC 500 mg twice each day.	Hematological malignancies	The proportion of patients that experience (NRM)	(139)

VC, vitamin C; DCR, disease control rate; NRM, non-relapse mortality.

#### 4.3.2 VC enhancing other anticancer therapies

Although VC or DHA monotherapy has certain anticancer effects, it should be noted that their *in vivo* stability is poor, their half-life is very short, and their therapeutic effects are limited. Therefore, high-dose VC anticancer treatment often requires the combination of other anticancer treatment methods, including immunotherapy, chemotherapy, radiotherapy, and photothermal therapy, to achieve the best effect.

##### 4.3.2.1 VC enhancing single anticancer therapy

Cimmino et al. (131) found that HDIVC induces DNA oxidation in leukemic cells, enhancing their sensitivity to PARP inhibitors and suppressing leukemic stem cell proliferation. The combination of VC with immune checkpoint inhibitors, such as CTLA-4 and PD-1/PD-L1 blockers, has shown promise in enhancing therapeutic responses across various cancers. Magrì et al. (100) demonstrated that high-dose VC, when paired with immunotherapy, can slow tumor growth in melanoma, colorectal, pancreatic, and breast cancers by promoting T-cell infiltration into the tumor microenvironment and enhancing CD8+ T cell cytotoxicity. Furthermore, Luchtel et al. (103) conducted a comprehensive analysis of the tumor immune microenvironment and demonstrated that VC significantly augmented the intratumoral infiltration of CD8+ T cells and macrophages. Their findings provide a compelling rationale for the investigation of combinations of high-dose VC with anti-PD1 agents in patients diagnosed with aggressive B-cell lymphoma, as well as in preclinical models of various other malignancies (103). High-dose VC was also applied as enhancer agent for EGFR-targeted therapy. Jung et al. (141) has shown that VC could enhance anti-EGFR efficacy of cetuximab by increasing ROS production in KRAS mutant colon cancer cell xenografts in a manner that was dependent on the expression levels of the SVCT2. While chemotherapy has improved tumor treatment outcomes, its adverse effects often lead to premature discontinuation, increasing the risk of recurrence and mortality. Ma et al. (142) reported that the intravenous administration of VC for cancer patients during chemotherapy can mitigate adverse reactions and enhance therapeutic efficacy. Although human trials are still limited in this study, their preliminary data suggest significant reductions in chemotherapy-related side effects. Additionally, Alexander et al. (143) found that intravenous VC sensitizes pancreatic cancer cells to radiotherapy, improving median overall survival (21.7 months vs. 12.7 months) and progression-free survival (13.7 months vs. 4.6 months) in patients with locally advanced pancreatic cancer. Furthermore, Ou et al. (144) conducted a phase I-II study showing that combining large doses of VC with radiofrequency deep heat therapy significantly improved the quality of life for patients with advanced non-small cell lung cancer (NSCLC). Overall, the integration of VC in cancer treatment regimens is gaining traction for its potential to enhance efficacy and reduce side effects.

##### 4.3.2.2 VC enhancing multiple anticancer therapies

Pharmacological doses of VC can effectively eradicate nearly all KRAS mutant cancer cells by modulating iron levels and oxidative stress (145). Additionally, a study published in PNAS revealed that VC supplementation enhances the efficacy of decitabine in treating myelodysplastic syndrome (MDS) and acute myeloid leukemia (AML), improving cancer cell inhibition and apoptosis (146). A combination therapy of fasting-mimicking diet (FMD), VC, and chemotherapy has proven to be the most effective for delaying tumor growth in xenograft models and extending survival in syngeneic models (147). Di Tano and Longo (147) demonstrated that curcumin and VC could inhibit the proliferation of triple-negative breast cancer cells, induced apoptosis, and downregulated MMP-2, MMP-9, and VEGF, thereby reducing cancer cell invasion and metastasis through modulation of the EGFR/PI3K/Akt signaling pathway. A clinical trial conducted from Ou et al. (148) involving 70 patients with advanced triple-negative breast cancer showed that the combination of curcumin and VC significantly improved progression-free survival (PFS) and overall survival (OS). A phase III clinical trial involving 442 colon cancer patients also achieved encouraging outcomes in the combined treatment of HDIVC (149). This trial demonstrated longer PFS in patients with KRAS mutation receiving HDIVC plus FOLFOX ± bevacizumab group rather than FOLFOX ± bevacizumab alone (9.2 vs. 7.8 months, HR 0.67; 95% CI, 0.50–0.91; *P* = 0.01).

#### 4.3.3 VC alleviating the side effects of cancer treatment

VC serves as a nutritional supplement that mitigates the adverse effects of cancer treatments rather than inducing toxicity. A retrospective study by Ou et al. (150) compared outcomes in advanced triple-negative breast cancer patients treated with gemcitabine and carboplatin alone versus in combination with VC. The study found that combining VC significantly reduced side effects such as bone marrow suppression, hair loss, nausea, vomiting, and constipation. Moreover, this combination enhanced the efficacy of chemotherapy, leading to an increase in progression-free survival (PFS) from 4.5 months to 7 months and overall survival (OS) from 18 months to 27 months. The percentage of patients surviving beyond 2 and 3 years rose to 54.3% and 14.3%, respectively, compared to 14.3% and 0% in the chemotherapy-only group. Additionally, Mikirova et al. (151) fund that HDIVC reduced inflammatory markers, including interleukin-1α, interleukin-2, interleukin-8, tumor necrosis factor-α, and C-reactive protein, and correlated with decreased tumor markers. A multicenter observational study further indicated that HDIVC improved cancer patients’ quality of life, providing significant relief from fatigue, pain, insomnia, and constipation after four weeks of treatment (152).

## 5 Discussion and outlook

VC, as a reductive essential nutrient, plays a significant role in both cancer prevention and treatment, alongside its normal physiological functions.

VC helps maintain homeostasis in the body through various mechanisms, thereby acting as an effective cancer preventative. Accordingly, a variety of biomarkers associated with the cancer-preventive effects of VC may be harnessed to identify high-risk cancer populations, encompassing individuals with genetic susceptibility, chronic inflammation, or perturbed oxidative stress status. These biomarkers may enable precise patient stratification by evaluating the potential benefits of VC intervention (151). For example, inflammatory and immune microenvironmental biomarkers, such as plasma concentrations of interleukin-1α, interleukin-2, interleukin-8, tumor necrosis factor-α, and C-reactive protein, combined with peripheral blood NK cell activity, can be leveraged to define a “high-risk inflammation-immunosuppression” subgroup, thereby informing targeted recommendations for VC supplementation. Metabolic and reparative pathway biomarkers, including the plasma GSSG/GSH redox ratio, may identify individuals who could experience a substantial reduction in cancer risk through VC-mediated metabolic regulation. Additionally, exposure-related biomarkers, such as urinary nitrosamines and their metabolites, can precisely characterize a “high-exposure high-oxidative damage” subgroup, prioritizing them for early intervention. However, several key issues remain unresolved: (1) Mechanisms of action. The specific molecular biological mechanisms by which VC prevents cancer are not fully understood. While many studies have confirmed its efficacy in cancer prevention, a systematic elucidation of these mechanisms *in vivo* is still lacking; (2) Optimal dosage. The ideal dosage of VC for cancer prevention has yet to be established. The current Chinese dietary reference intake recommends 200 mg/day for preventing non-communicable chronic diseases. However, high doses may pose risks of serious side effects, particularly in individuals with renal insufficiency, G6PD deficiency, hemochromatosis, and hypercoagulability.

Moreover, VC exhibits anticancer properties through several pathways, including inducing oxidative stress, regulating epigenetic expression, modulating HIF-1 activity, reducing tumor cell malignancy, and enhancing tumor sensitivity to treatments. Consequently, biomarkers associated with the anticancer efficacy of VC offer potential for predicting response to VC combination therapies, monitoring therapeutic efficacy, and elucidating mechanisms of drug resistance (90–94). TME parameters such as hypoxia and redox status, reflected by *HIF-1*α protein expression and glutathione (GSH) content in tumor tissue, can inform clinical decisions regarding VC monotherapy versus combination strategies. Indicators of immunosuppression and immune activation, including PD-L1 expression and CD8+ T cell infiltration within tumors, are relevant for therapeutic approaches combining VC with immune checkpoint inhibitors (100, 103). Markers of energy metabolism and the acidic TME, exemplified by tumor interstitial fluid pH, may identify patients likely to benefit from VC monotherapy or VC-combined metabolic targeting therapies. Furthermore, dynamic monitoring biomarkers, such as serum MMP-9, enable real-time assessment of VC combination therapy efficacy and facilitate treatment regimen adjustments. However, the following questions remain: (1) The doses required to achieve anticancer effects are significantly higher than physiological concentrations. While physiological levels can exert epigenetic effects, it is unclear if anticancer treatment should be tailored based on specific treatment objectives; (2) The selectivity of VC’s antitumor effects is still enigmatic. High-dose VC can eliminate tumor cells while sparing normal tissue, yet only certain tumor types respond. The molecular mechanisms behind this selectivity are still unknown; (3) The interplay among VC’s anticancer mechanisms is not well understood. It remains to be determined whether novel mechanisms are involved, how multiple reported mechanisms interact, and how they synergize with other anticancer strategies to maximize efficacy; (4) The safety concerns are associated with VC’s biological instability, influenced by pH, light exposure, and temperature. High-dose intravenous VC may lead to severe side effects, including renal failure in patients with kidney issues, hemolysis in those with G6PD deficiency, iron overload in hemochromatosis patients, and thrombosis in cancer patients.

In summary, while VC has shown promise in cancer prevention and treatment research, most studies remain foundational, primarily consisting of low-level evidence such as case reports and phase I/II clinical trials. There is a notable lack of long-term, multicenter, large-scale, and prospective standardized research. Additionally, inconsistencies in clinical data have emerged, with some findings suggesting that the disadvantages of using VC in anticancer treatment may outweigh its benefits, potentially undermining its effectiveness in cancer prevention. Therefore, further research on VC in the context of cancer prevention and treatment is crucial to uncover new insights and identify alternative targets for effective anticancer strategies. Based on emerging technologies, the integration of multidimensional biomarker panels, incorporating artificial intelligence-enhanced metabolomics, transcriptomics, and proteomics, may also further enhance the accuracy of patient risk stratification for cancer prevention and therapy using VC. Specifically, establishing a VC-associated cancer prevention and therapeutic biomarker database through multicenter cohort studies is essential, as it may rapidly facilitate large-scale clinical translation of VC applications in oncology.
